# Macro-Mesoscale Mechanical Properties of Basalt-Polyvinyl Alcohol Hybrid Fiber-Reinforced Low-Heat Portland Cement Concrete

**DOI:** 10.3390/polym15030621

**Published:** 2023-01-25

**Authors:** Yu Zhang, Yuanxun Zheng

**Affiliations:** 1 Yellow River Laboratory, Zhengzhou University, Zhengzhou 450001, China; 2 School of Water Conservancy and Civil Engineering, Zhengzhou University, Zhengzhou 450001, China

**Keywords:** low-heat Portland cement concrete, basalt fiber, polyvinyl alcohol fiber, mechanical properties, mesoscopic numerical analysis

## Abstract

To investigate the mechanical properties of hybrid basalt fiber (BF) and polyvinyl alcohol fiber (PVAF)-reinforced low-heat Portland cement concrete (LHPCC), two groups of concrete were formulated. The BF and PVAF were equal in the first group, and the total fiber contents were 0–0.4%. The second group was the total fiber content of 0.3% and the occupancy of BF in the hybrid fiber of 0, 25%, 50%, 75%, and 100%. Two groups of concrete were tested for compressive, splitting tensile, and flexural strengths to illustrate the enhanced mechanism of the mechanical properties of LHPCC by hybrid fiber. The best mechanical property enhancement was achieved when BF and PVAF were in equal proportions and the fiber content was 0.3%. Meanwhile, the test results for the mechanical properties were also compared with the predicted values of ACI 318 and Eurocode 2. Moreover, the hybrid BF-PVAF-reinforced concrete was regarded as a three-phase composite material consisting of fiber-reinforced mortar, coarse aggregate, and an interfacial transition zone. The axial compressive and splitting tensile strengths, damage processes, and mechanical strengthening mechanisms of concrete were investigated for different total fiber content at equal ratios of BF and PVAF, and the results were compared with the macroscopic mechanical test findings. The results show that the conclusion of the meso-simulation matches well with the test. Finally, the effect of aggregate and hybrid fiber content on the mechanical properties of LHPCC was predicted by a simulation. The results of this study can provide references for future mechanical modeling, performance studies, and practical engineering applications of LHPCC.

## 1. Introduction

Concrete is used in many civil engineering fields because it is easy to obtain materials, reasonably priced, and convenient to construct. Ordinary Portland cement (OPC) is one of the typical raw materials for concrete, but it has the following defects: high hydration heat, poor corrosion resistance, and wet and heated curing, so it is not suitable for pouring hydraulic high-volume concrete. Relative to OPC concrete, low-heat Portland cement concrete (LHPCC) has a slower early temperature rise and high late strength, which can effectively reduce the difficulty of temperature control and crack prevention in mass concrete [1,2,3,4]. However, LHPCC still suffers from poor tensile strength, slight elongation, and high brittleness. Therefore, it is of great significance to further improve the performance indexes of LHPCC and to study the economical and effective methods for toughening and crack resistance of concrete for the application and promotion of LHPCC [5].

The study shows that the addition of fibers does not affect the characteristics of the concrete and can bring its material properties for toughening and crack resistance. Common fibers include steel fibers [6,7,8], synthetic fibers [9,10], and natural fibers [11,12]. Among them, short-cut basalt fibers (BF) have a great bonding force of base material, high strength, and corrosion resistance, and can be adapted to various environmental conditions. It was shown [13,14,15,16] that BF can improve the tensile strength, fracture modulus, toughness, and deformation resistance of concrete. Zheng et al. [16] concluded that the mechanical properties of concrete were effectively improved when BF at 10–20 μm in filament diameter, 12–20 mm in length, and 1% in volume fraction by combing the study of mechanical properties. At the same time, the synthetic fiber represented by polyvinyl alcohol fiber (PVAF) has excellent hydrophilicity, impact resistance, cohesiveness, and dispersion. So it has been widely used in concrete. Khan et al. [17] found that adding PVAF provided concrete with higher stiffness and deflection hardening behavior and improved the flexural toughness of high-strength concrete. The fiber aspect ratio of 45 provided maximum strength for concrete at 28 d, which was improved by 2.35% compared to plain concrete. Zhang et al. [18] derived that the splitting tensile and flexural strengths of ultra-high-strength concrete were improved by 35.2% and 26.7% when the PVAF content was 1.5%.

The same type of fiber has a limited effect on improving the mechanical properties of concrete. However, the concrete is reasonably hybrid with different kinds of fibers and can fully combine with the cement matrix, which effectively improves the mechanical properties of concrete [19]. Almusallam et al. [20] and Abadel et al. [21] investigated the mechanical properties of hybrid fiber-reinforced concrete (HFRC) produced with three fibers: steel, Kevlar, and polypropylene. The results showed that the admixture of hybrid fibers improved compressive strength, fracture properties, and toughness. Modulus of rupture, splitting tensile, and direct tensile strengths were increased by 76–159%, 60–90%, and 56–75%, respectively. Krishna et al. [22] investigated the effect of steel, polypropylene, and sisal fibers on compressive strength at high temperatures to replace individual steel fiber-reinforced concrete to improve the residual hardening properties and fire resistance. Khan et al. [23] investigated the strengthening index of multi-scale hybrid fiber concrete. The results showed that the best mechanical properties of the concrete were obtained with a BF content of 0.8%, CaCO_3_ whiskers of 1%, and steel fibers of 0.25%. El Ouni et al. [24] showed through a study that the flexural strength of recycled aggregate concrete increased by 26% compared to normal concrete when reinforced with 0.85% steel and 0.15% polypropylene fibers. The impermeability has also been improved.

High elastic modulus and ductility of fibers that are hybrid in a particular ratio can generally significantly improve the tensile and ductile properties of concrete, which can enhance the initial cracking, strength, ductility, and toughness of concrete with the property of fibers [25]. Additionally, the hybrid of the high elastic modulus of BF and the high ductility of PVAF could achieve a positive modification effect on the mortar matrix, thus achieving a greater degree of concrete performance [26,27]. In recent years, the research results of hybrid PVAF and BF-reinforced concrete have been carried out successively. Özkan et al. [26] added BF and PVAF with a total content of 2% to concrete. As the proportion of BF in the hybrid fiber increased from 0 to 100%, the porosity decreased by 35.08%, the water absorption decreased by 44%, the capillary suction coefficient decreased, and the hardened density value increased by 28%. Jalal et al. [28] studied that hybrid BF and PVAF improved the ductility of concrete and prolonged the load-deflection curve. The initial cracking toughness, flexural toughness, and maximum deflection increased by 54%, 8%, and 74% when both BF and PVAF contents were 0.15%. In summary, studies on hybrid BF and PVAF-reinforced concrete have mainly focused on plain concrete, and it is necessary to study LHPCC further. At the same time, past research on LHPCC focused more on the ratio test, mechanical properties, physical properties, and durability [29,30,31]. In contrast, the research on the mechanism of fiber toughening and crack resistance for LHPCC still needs to be systematic.

In recent years, finite element analysis has become an essential tool for studying material properties. Scholars have tried to use this method to simulate and analyze the mechanism of performance enhancement of concrete by fibers [32,33,34,35]. Sun et al. [36] concluded that increasing the content of BF could effectively increase the peak load and fracture energy of concrete from three-point flexural tests and numerical simulations of prefabricated notched beams. Naderi et al. [37] demonstrated the fracture process of steel fiber-reinforced concrete during uniaxial tensile and compression based on the 3D mesoscale structural features of steel fiber-reinforced concrete, including fibers, aggregates, mortar, interfacial transition zones, and voids. The flexural test of the concrete beam was simulated by Khan [38] at the optimum fiber content (0.8% for steel and 0.1% for polypropylene fibers). The maximum flexural capacity of the model was 88.2 kN, the maximum deflection was 5.4, and the first crack appeared at 35.84 kN. The results of the above studies show that the addition of fibers in the process of concrete hardening can reduce the number and scale of plastic cracks and microcracks, improve the continuity of the concrete material media, and ultimately improve the overall performance of concrete. However, the current mesoscale simulation study of LHPCC by the finite element method is still inadequate.

In this paper, the hybrid BF and PVAF-reinforced LHPCC were studied to improve the performance of concrete in multiphase, multi-structure, and multi-level by the complementary combination of two fibers to obtain more significant mechanical properties than those under single fiber conditions. Based on this, this paper presents a comprehensive investigation of the workability, compressive, splitting tensile, and four-point flexural properties of hybrid fiber-reinforced LHPCC. The hybrid BF-PVAF-reinforced LHPCC model at the mesoscale level was also established using Abaqus finite element analysis software. The damage evolution laws of LHPCC with various hybrid fiber contents and the macro-mechanical test results were compared and analyzed. The two fibers in this study are hybridized to improve the deformability and toughness of LHPCC, which alleviates the disadvantages of its low toughness and higher brittleness. At the same time, a practical and relatively simple numerical model is provided for the analysis of hybrid fiber-reinforced LHPCC. The development of this study can provide a new way for the technical application of LHPCC in water conservancy projects.

## 2. Test Scheme

### 2.1. Performance of Raw Materials

**Figure 1:**
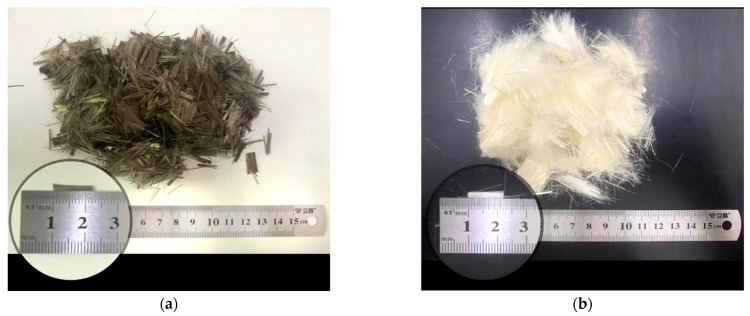
Appearance and length of BF and PVAF. (**a**) BF and (**b**) PVAF.

The cement is P·LH42.5 produced by Jiahua Jinping Special Cement Co., Ltd. from Sichuan, China. The test results meet the requirements for “Moderate heat Portland cement, Low heat Portland cement, and Low heat Portland slag cement” (GB200-2003), and the test results for each performance index of the cement are shown in Table 1. The fine aggregate is natural river sand with a fineness modulus of 2.67, which belongs to the range of medium sand. Coarse aggregate is continuously graded gravel with particle sizes of 5–20 mm. The properties of coarse and fine aggregates conform to the requirements in “Pebble and Crushed Stone for Construction” (GB/T14685-2011), “Sand for Construction” (GB/T 14684-2011), and “Specifications for Hydraulic Concrete Construction” (DL/T5144-2015). The performance indexes of coarse and fine aggregates are shown in Table 2, and the sieving test results are shown in Table 3. The coal fly ash is produced by Guodian Xuanwei Power Generation Co., Ltd., in Xuanwei, China. with grade I and 35% admixture, and the inspection results for physical quality are presented in Table 4. The admixtures used in the test are a JM-II retardation-type high-efficiency water-reducing agent produced by Jiangsu Subot and an air-entraining agent produced by Zhejiang ZB-1G. The short-cut BF is produced by Haining Anjie Composites Company, and PVAF is produced by Japan Kuraray Company, with the appearance and length of fiber shown in Figure 1 and the performance index shown in Table 5. The dispersant in PVAF is polyacrylamide, which can reduce the surface tension of the mixed solution of dispersant and water and improve the wettability of fiber while not reacting chemically with cement.

### 2.2. Concrete Mix Proportion Design

According to the available research results, the design principles of concrete cubic compressive, tensile, and flexural resistance can be used when the fiber content is low. This method is approximately the same as the design method for the plain concrete mix proportion [39]. According to the experience of fiber-reinforced concrete mix proportion design combined with the mix proportion design method in ACI 211.1 [40], the preliminary mix proportion design of concrete was carried out. The proportion of each component was adjusted after trial mixing to determine the amount of each component. The basic mix proportions of LHPCC designed for the test are shown in Table 6. In order to investigate the effect of total fiber content and the proportion of hybrid fibers on concrete, different amounts of fibers were added directly to the concrete mix (the number of fibers was calculated as a volume fraction) without changing other components of concrete. They are presented in Table 7.

### 2.3. Preparation and Maintenance of Specimens

**Figure 2:**
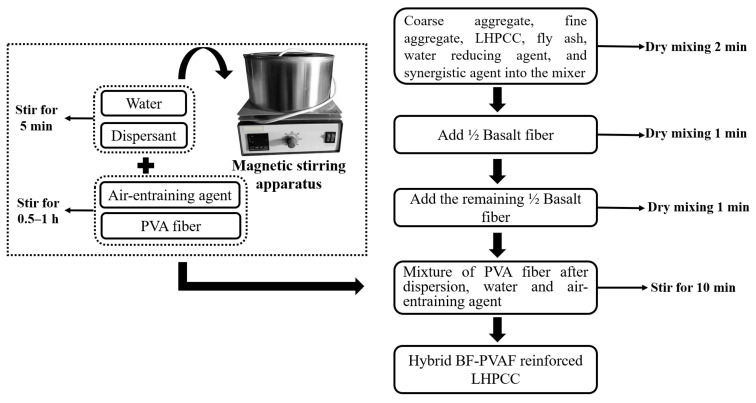
Fabrication process of hybrid BF-PVAF-reinforced LHPCC.

Dry mix coarse aggregate, fine aggregate, low-heat Portland cement, coal fly ash, and a water-reducing agent in the mixer for 2 min to get the aggregate mixture. Add half of the short-cut BF and dry mix for 1 min, then add the other half of the BF and dry mix for 1 min. As the fiber-reinforced concrete is prepared, PVAF is easy to cluster. So, the dispersion of fibers should be fully considered to ensure that the fibers are not clustered, not in bundles, and evenly dispersed in the concrete. After several trial mixes, PVAF was used to add the technology: mix the dispersant, PVAF, air-entraining agent, and the appropriate amount of water, then stir in the magnetic spectroscopy stirrer for 30 min–1 h. After PVAF was pre-dispersed, it was added to the aggregate mix mentioned above and continued to be stirred for 10 min to obtain the hybrid BF-PVAF-reinforced LHPCC mix, which was then filled into the mold. The preparation steps are shown in Figure 2.

Since the setting time of low-heat Portland cement is slower than that of ordinary cement, the specimens were placed indoors for 48 h and then removed, during which the room temperature was ensured to be 20 ± 5 °C. Subsequently, the specimens were moved into the standard curing room for 28 d. The temperature was 20 ± 2 °C, and the relative humidity was above 95%. The specimens in this test are non-standard specimens; the size of the compressive and splitting tensile specimens is 100 × 100 × 100 mm, and the size of the flexural specimens is 100 × 100 × 400 mm.

### 2.4. Test Methods

**Figure 3:**
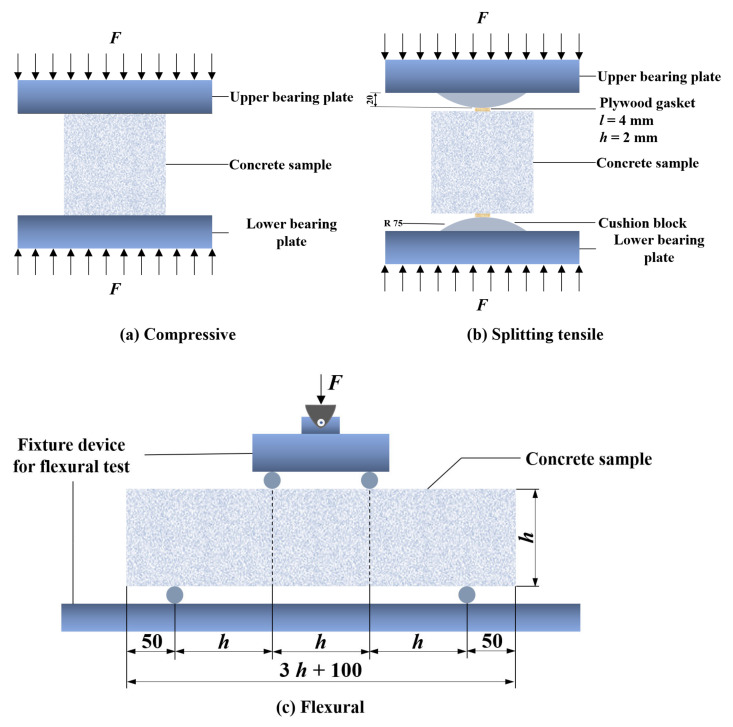
Schematic diagram of the tests on basic mechanical properties. (**a**) Cube compressive test; (**b**) Splitting tensile test; (**c**) Flexural test.

The slump test was carried out according to ASTM C143 [41] to evaluate the workability of the concrete. Compression tests were carried out according to ASTM C39 [42]. Flexural tests were carried out according to ASTM C1609 [43]. Splitting tensile tests were carried out according to Chinese standard GB/T 50081-2002 [44]. The instrument used was an SHT4106-G microcomputer-controlled electro-hydraulic servo universal testing machine. The specific operation methods of the tests are shown in Figure 3. Rocco et al. [45] showed that the plywood gasket width affects the splitting tensile strength of concrete. The effect of gasket width on the splitting tensile strength of concrete can be ignored when the proportionality of the gasket width to the side length of the cubic concrete is less than 4%. In this study, the plywood gasket width was 4 mm, and the height was 2 mm. The loading rates are: 0.3–0.5 MPa/s for compression, 0.04–0.06 MPa/s for splitting tensile, and 0.05–0.08 MPa/s for flexural were loaded at steady rates.

## 3. Test Results and Analysis

### 3.1. Slump

**Figure 4:**
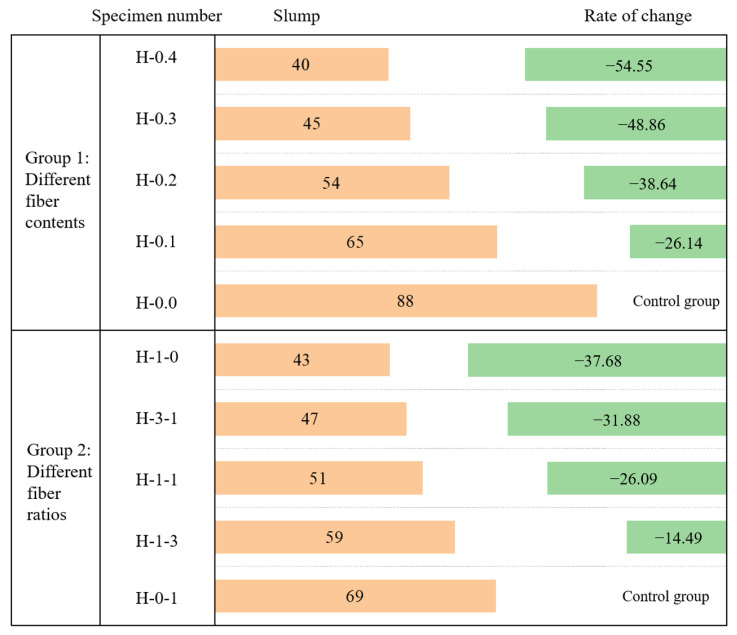
Effect of fiber content and ratio on the slump of LHPCC.

In this study, the effects of total fiber content and the ratio of two fiber hybrids on slump were investigated separately. The results of the workability tests are illustrated in Table 8 and Figure 4. The slump of freshly mixed concrete gradually decreased with the increase in total fiber content in the first group. When the fiber content was 0.4%, the slump decreased to 54.55% compared with the control group. The total fiber contents were 0.3% in the second group, and the higher the BF content in the mixture, the smaller the slump, and the slump of PVAF-reinforced concrete was 37.68% higher than that of BF-reinforced concrete. That is, the total fiber content has a more significant effect on the slump than the proportions of the two fibers. Compared to plain concrete, the addition of fibers hinders the sinking of the mix. At the same time, both BF and PVAF have water absorption, which reduces the amount of water used in the mix and lowers the water-cement ratio, decreasing the workability of the concrete.

### 3.2. Compressive Strength

In this paper, two groups of tests were set up for mechanical property testing. Group 1: BF and PVAF were selected to be hybridized at a ratio of 1:1, and the total fiber content was 0%, 0.1%, 0.2%, 0.3%, and 0.4%, as presented in Table 9. Group 2: The total fiber contents were 0.3%, and the proportion between the two fibers was 0:1, 1:3, 1:1, 3:1, and 1: 0, i.e., BF to a total fiber volume of 0%, 25%, 50%, 75%, and 100%. The test results are shown in Table 10.

**Figure 5:**
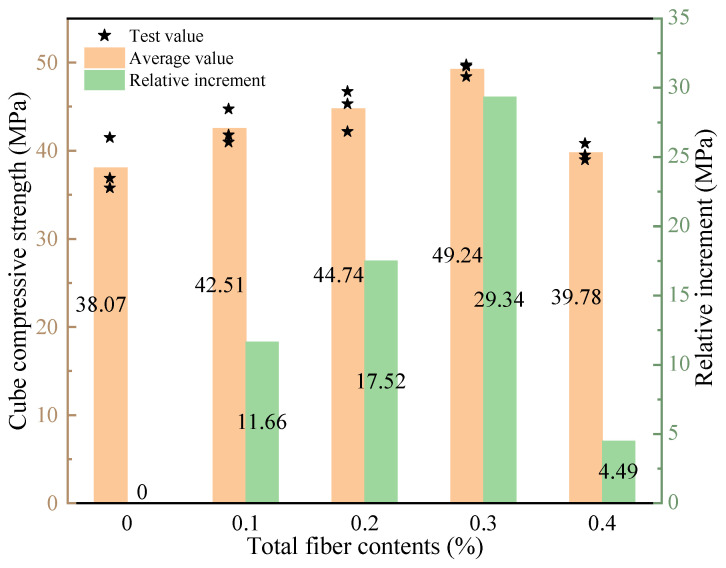
Compressive strength of LHPCC with different total fiber content.

The 28-d compressive strength of the concrete at various total hybrid fiber contents is presented in Figure 5. The compressive strength shows a trend of growth and then decreases with the increase of total hybrid fiber content, and the trend of concrete compressive strength changes more obviously when the fiber contents are in the range of 0.2–0.4%. When the fiber contents reached 0.3%, the compressive strength reached a maximum value of 49.24 MPa, which was 29.34% higher than the control group. This is due to the different diameters of BF and PVAF, and they can effectively fill the voids of different scales in concrete, prevent the development of microcracks, reduce the internal porosity, and improve the compressive strength of concrete. When the total content reaches 0.4%, the compressive strength decreases significantly but is still higher than the reference concrete. This is because it is easy to produce clustering phenomena inside the concrete, thus forming weak surfaces.

**Figure 6:**
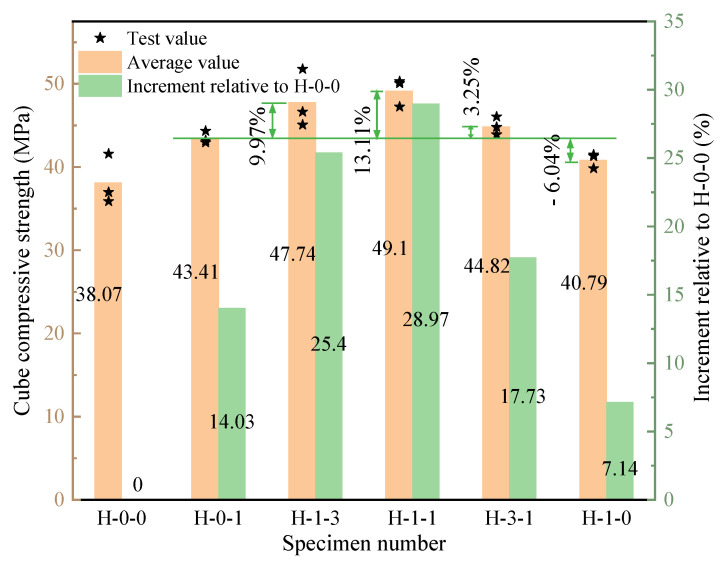
Compressive strength of LHPCC with different fiber hybrid proportions.

Figure 6 shows the compressive strength of LHPCC at different fiber hybrid proportions. The concrete of the H-0-0 group without fiber was added as the control group to study the increment of the compressive strength of concrete with different fiber hybrid proportions compared to H-0-0. As the BF volume occupancy rose from 0 to 50%, the compressive strength improved remarkably, reaching its maximum strength when the BF occupancy was 50%. The compressive strength was improved up to 28.97% in comparison with the control group and by about 13.11% with the single BF-reinforced LHPCC. That is, when the hybrid proportion of BF and PVAF is 1:1, the compressive strength enhancement of hybrid fiber-reinforced concrete is the highest. When the BF content exceeded 50%, the compressive strength gradually decreased, and when the content reached 100%, the compressive strength decreased by 6.04% compared with the H-0-1 group but was still higher than the control group by 7.14%.

### 3.3. Splitting Tensile Strength

**Figure 7:**
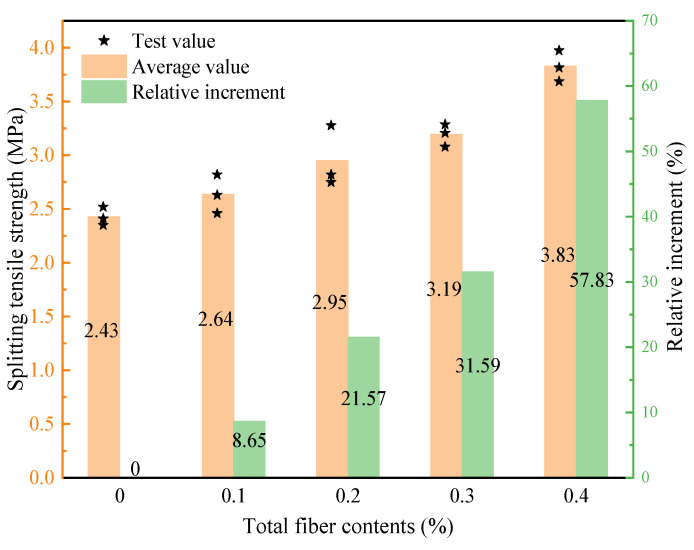
Splitting tensile strength of LHPCC with different total fiber content.

The 28-d splitting tensile strengths of LHPCC with different total hybrid fiber contents are shown in Figure 7. The splitting tensile strength of LHPCC with 0.1%, 0.2%, 0.3%, and 0.4% of total fiber content increased by 8.65%, 21.57%, 31.59%, and 57.83% for the base concrete. The splitting tensile strength increased with the total fiber content. Moreover, the effectiveness of hybrid fibers was more pronounced in the splitting tensile strength of LHPCC than the compressive strength.

**Figure 8:**
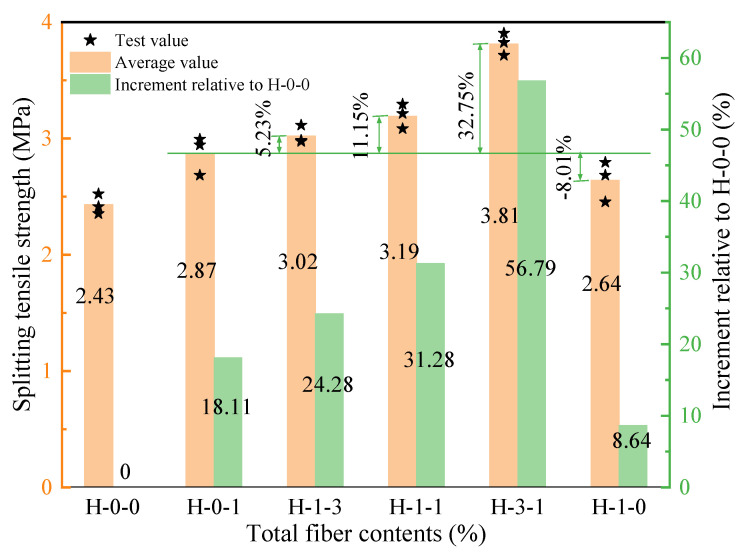
Splitting tensile strength of LHPCC with different fiber hybrid proportions.

Figure 8 shows the splitting tensile strength of LHPCC with different fiber hybrid proportions for a total fiber content of 0.3%. Moreover, the H-0-0 group without fiber was added as the control group to study the increment of the splitting tensile strength of concrete with different fiber hybrid proportions compared to the control group. It can be seen that the splitting tensile strength increases continuously when the BF occupancy in both fibers increases in the range of 0–75%. When the ratio of BF to PVAF reaches 3:1, the splitting tensile strength of LHPCC has a maximum value of 3.81 MPa, which is improved by 56.79% compared to the H-0-0 group and by 32.75% compared to the H-0-1 group. When the volume occupancy of BF reached 100%, the splitting tensile strength turned out to be significantly reduced and was lower than PVAF-reinforced LHPCC by 8.01% but still higher than the control group by 8.64%. Since PVAF has a low elastic modulus and high toughness compared to BF, and the elastic modulus has more influence on concrete than toughness, the best value of splitting tensile strength was achieved at the hybrid proportion of 3:1. When 100% of the BF is in concrete, the elastic modulus is effectively increased, but with less PVAF toughness influence, the strength is rather unfavorable.

### 3.4. Four-Point Flexural Strength

**Figure 9:**
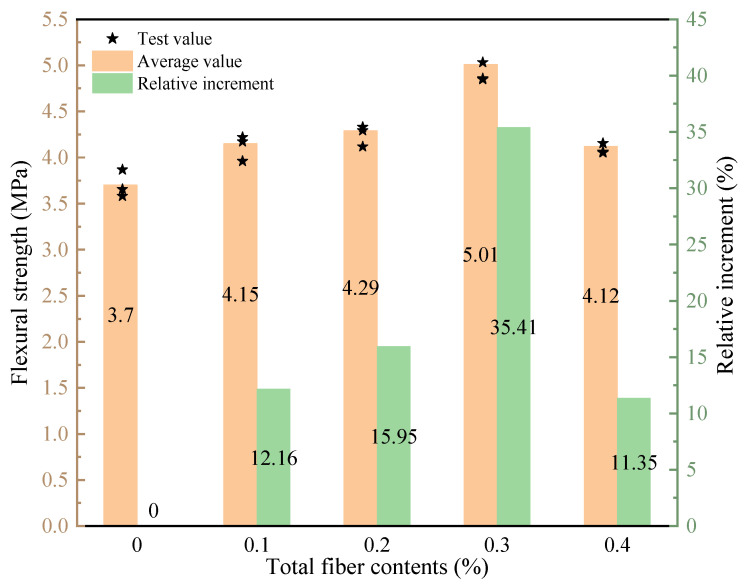
Flexural strength of LHPCC with different total fiber content.

The effect of total hybrid fiber contents on the flexural strength of LHPCC is shown in Figure 9. The hybrid of BF and PVAF could effectively improve the flexural strength of LHPCC specimens, and the specimens with fiber contents of 0.3% had the most significant flexural strength enhancement, which increased by 35.41% compared with the control concrete. The flexural strength started to decrease with a further increase in the content of hybrid fibers. The flexural strength value at 0.4% content was close to that at 0.1% but still higher than the control group. This is because the function of cracking resistance and the wall effect of both fibers differ from normal concrete, so they effectively slow down crack development in LHPCC.

**Figure 10:**
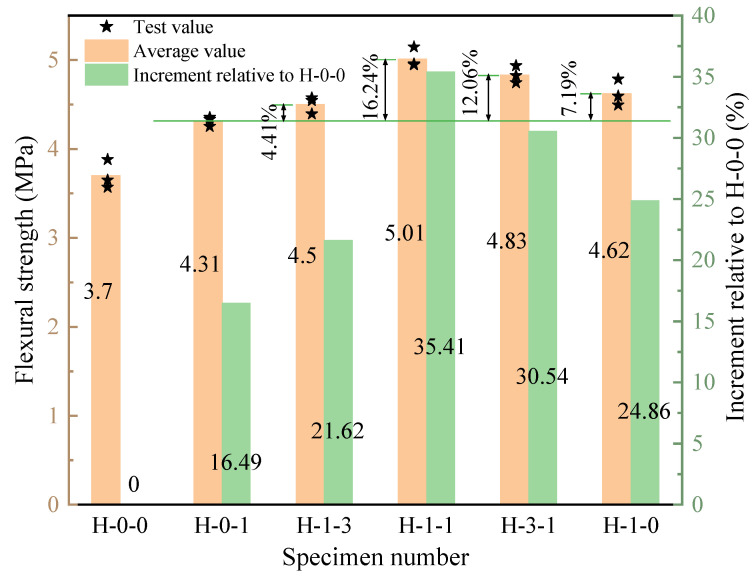
Flexural strength of LHPCC with different fiber hybrid proportions.

The flexural strength of LHPCC in various fiber hybrid proportions is shown in Figure 10, and the total fiber content is 0.3%. The H-0-0 control group without fiber was also set to study the increment of the four-point flexural strength of concrete with different fiber hybrid proportions compared to the control group. It can be seen that the flexural strength with the addition of fibers in all five groups has increased in different degrees relative to the control group. As the proportion of BF in the hybrid fibers increased, the flexural strength presented the tendency to improve and then reduce, and the optimum value existed. The flexural strength was low when PVAF was added individually and increased gradually as the ratio of BF to PVAF changed from 0:1 to 1:1. The flexural strength reached its best value when the BF occupancy reached 50%, which increased by 35.41% compared with the control group and 16.24% compared with PVAF-reinforced concrete. After the BF occupancy exceeded 50%, the flexural strength gradually decreased. When the BF contents are 75%, the flexural strength is 3.59% lower than the maximum. When the BF occupancy reaches 100%, the flexural strength is about 7.78% lower than the maximum, which is still higher than the PVAF-reinforced concrete by 7.19%.

### 3.5. Comparison of Test Values with Codes Predictions

**Figure 11:**
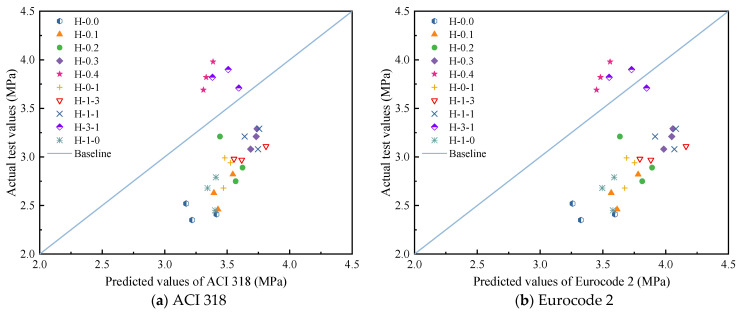
Comparison of test values and code predictions values of splitting tensile strength.

**Figure 12:**
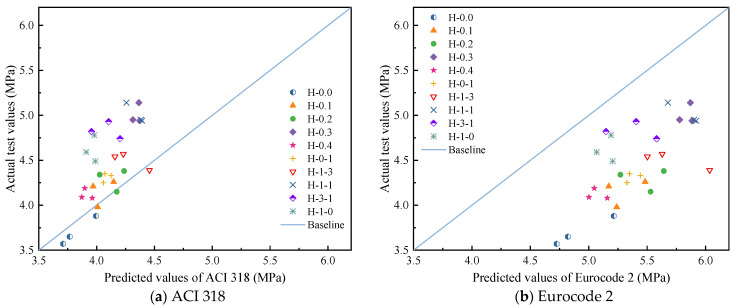
Comparison of test values and code predictions values of flexural strength.

This paper compares the test values with the expressions recommended in the existing American code ACI 318-11 [46] and Eurocode 2 (2004) [47] to investigate the applicability of the two codes for hybrid fiber-reinforced LHPCC, as shown in Table 11. The predicted splitting tensile and flexural strengths are compared with the test values, as shown in Figure 11 and Figure 12. It can be seen that for the splitting tensile strength of LHPCC, the predicted values of both codes are higher than the actual strength values. However, the predicted values are lower for the fiber content of groups H-0.4 and H-3-1. This indicates that the splitting tensile strength of LHPCC is relatively low compared to normal concrete and can gradually approach the predicted value by adding hybrid BF and PVAF. For flexural strength, the predicted value of ACI 318 was closer to the test value, but the overall predicted value was low. On the contrary, the predicted flexural strength of Eurocode was significantly high and differed from the test value.

### 3.6. Synergy Effect of Hybrid Fibers in Concrete

**Figure 13:**
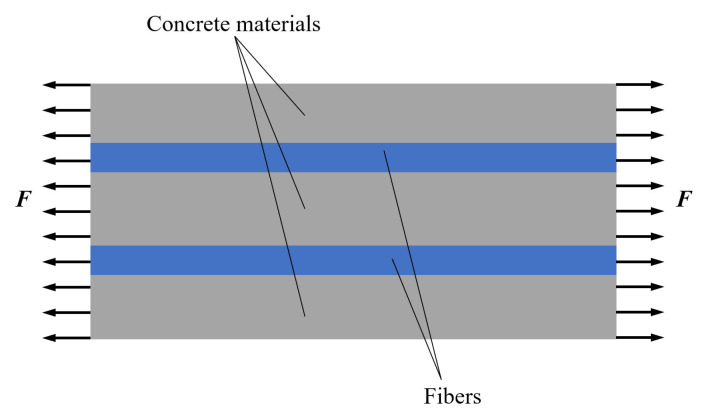
Composite materials force diagram.

Composites theory regards fiber-reinforced concrete as a two-phase system composed of fibers and concrete, and the sketch is shown in Figure 13. Therefore, based on this study, it is only by fully understanding the intrinsic mechanisms of hybrid BF and PVAF in LHPCC that the synergistic effect between concrete and fiber can be fully exploited.

#### 3.6.1. Toughening Mechanism

Theoretical analysis of the reasons for the toughening of concrete by fibers can suggest theoretical support for the effects of fibers and the development of new materials. The enhanced toughness of hybrid BF and PVAF-reinforced LHPCC compared to plain concrete is due to the synergistic mechanism of BF and PVAF in concrete. BF has a relatively large elastic modulus and can mainly assume the bridging role at the crack. In contrast, PVAF has better deformation performance and ductility, which can consume more energy through its deformation during the process of crack expansion. The explicit division of labor and synergistic work of the two fibers suppress cracking throughout the whole process and ensure an increase of LHPCC toughness. Meanwhile, as PVAF has better dispersion in cement than BF, it can also help BF disperse evenly after hybridization and enhance the bonding effect between BF and cement, which enables the fibers to consume further energy in the pull-out process.

#### 3.6.2. Crack Resistance Mechanism

The study of the crack resistance mechanism of hybrid fiber-reinforced LHPCC is the theoretical basis for its application to concrete structures. LHPCC in the mixing and pouring process, BF and PVAF in the concrete random distribution and lap, forming a mesh structure to prevent the sinking of coarse and fine aggregates. The hybrid of various diameters of BF and PVAF can enable the compaction of LHPCC and mitigate the development of cracks [48]. Meanwhile, the surface of both fibers collects a large number of hydration products, which block the channels for water loss and improve the crack resistance of the matrix by reducing the probability of cracks.

The effect of BF and PVAF on the matrix of LHPCC exists throughout the hardening process of concrete. The lower elastic modulus of PVAF retards the formation and expansion of microcracks in the early stage of LHPCC hardening. The high elastic modulus of BF allows the fibers to form a load-bearing framework in the matrix, which has a more pronounced inhibition of crack expansion in hardened concrete [49].

#### 3.6.3. Mechanical Performance Improvement Mechanism

From the studies mentioned above, it is shown that the appropriate amount of BF and PVAF were hybrid to improve the mechanical performance of LHPCC effectively.

**Figure 14:**
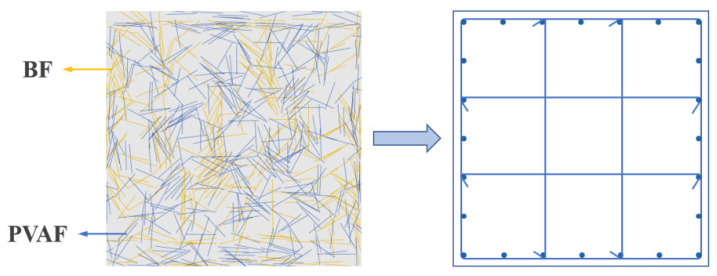
The working effect of force-transmitting fiber micro-rebar mesh is equivalent to that of hooped reinforcement.

Firstly, since the dispersion of PVAF in concrete is better than that of BF, BF tends to cluster in concrete when the fiber contents are larger, thus generating weak structural zones. The excellent dispersion of PVAF can form a force-transmitting fiber micro-rebar mesh with BF, restraining the small cracks in the concrete due to shrinkage or water evaporation. These fiber-meshes exert a hoop-like working effect when the concrete specimen is subjected to axial pressure, increasing the compressive strength of the concrete by providing lateral constraint force, as shown in Figure 14. As the fiber contents increase, the more fiber meshes are formed, and the more the compressive strength increases are pronounced [50].

**Figure 15:**
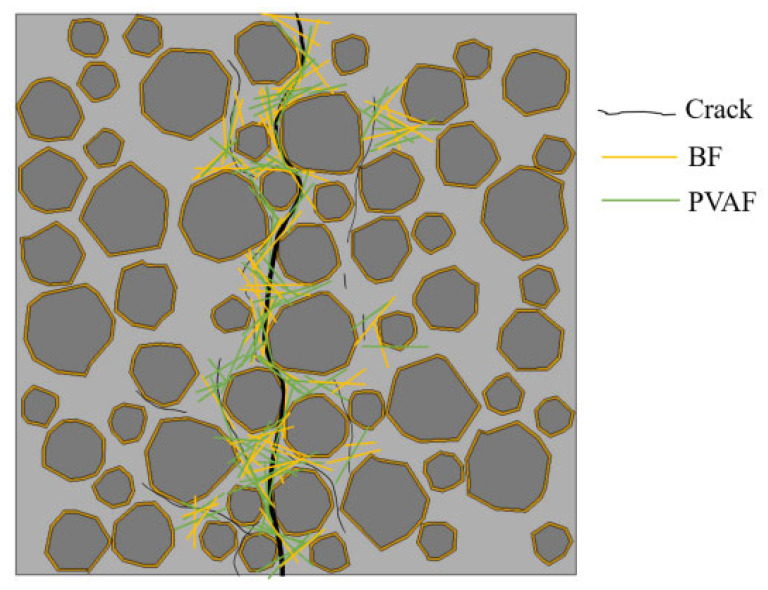
Effect mechanism of hybrid fibers on splitting tensile properties of concrete (only fibers at cracks are shown).

Secondly, tensile and plastic strength during the early hardening of concrete were increased by the addition of BF, which can reduce the porosity inside concrete by reducing of early shrinkage cracks. The addition of PVAF enhances the bonding between the hybrid fibers and the cement. It also improves the elastic modulus of the concrete matrix at the early stage of hardening. The better bonding between the hybrid fibers and the cement reinforced the above effects. In addition, the combination of the two fibers improves the interface transition zone (ITZ) [51] and reduces the stress concentration at the tip of the microcrack, which further improves the tensile strength of LHPCC. The mechanism of hybrid fibers on the splitting tensile properties is shown in Figure 15, where fibers penetrate macroscopic and microscopic cracks to prevent crack expansion, and some fibers fill the ITZ and enhance its mechanical strength.

**Figure 16:**
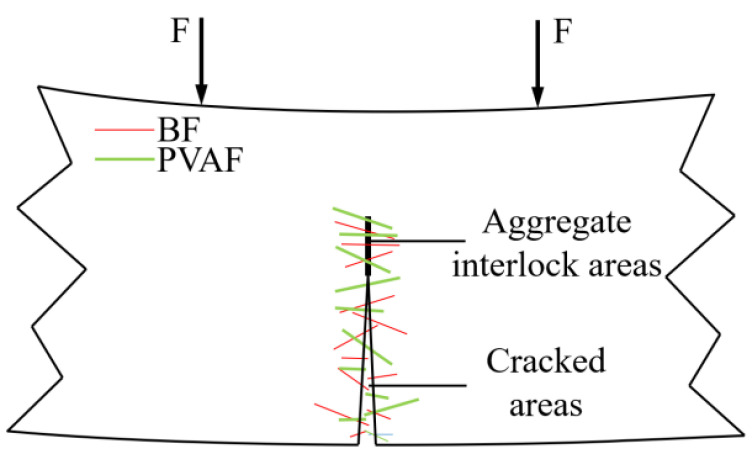
The effect mechanism of hybrid fiber on concrete flexural damage.

Finally, BF assumes the bridging role at the cracks when concrete is subjected to flexure and tension. At the same time, PVAF absorbs a large amount of energy through its deformation, and the synergistic work of the two fibers significantly enhances the flexural performance of LHPCC. The action forms of BF and PVAF in the flexural specimens are shown in Figure 16.

#### 3.6.4. Economic Efficiency

From an economic point of view, hybrid fiber also has certain advantages. The high tensile strength and elastic modulus of PVAF are good for toughening and crack resistance in concrete, but the cost is high. BF is economical, but toughening and crack resistance effects are relatively weak compared to PVAF. Therefore, the hybrid of BF and PVAF can increase the strength, toughness, and crack resistance of concrete with less cost, which is good for improving the brittle characteristics of concrete.

## 4. Mesoscale Simulation Analysis of Hybrid Fiber-Reinforced LHPCC

**Figure 17:**
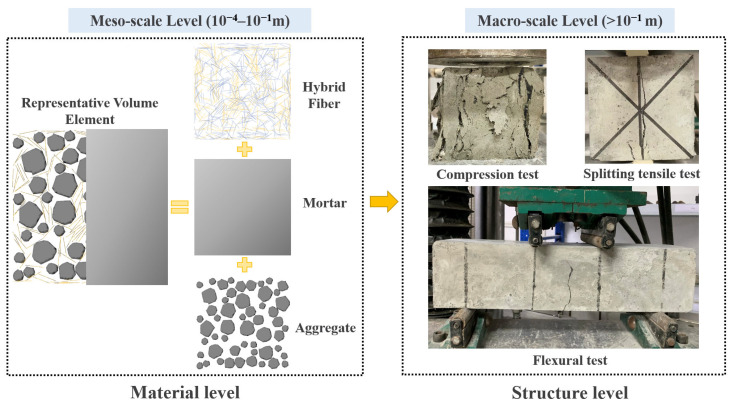
Macro-mesoscale analysis of hybrid fiber-reinforced LHPCC structure.

Based on the macro-mechanical study, the mesoscale level analysis of BF and PVAF-reinforced LHPCC is used to laterally respond to the failure mechanism of specimens at the structural level from the change mechanism in each component performance at the material level. Establishing an accurate link between the macroscale and mesoscale mechanical tests, as shown in Figure 17, this study is based on the foundation of the finite element analysis method, and a numerical model of multiphase mesoscale mechanics of concrete with random aggregates is established. It includes the generation and placement of random aggregates, the three-phase medium pretreatment process of aggregates, the mortar interface, and the constitutive relations with damage determined to build a mesoscale model based on the parameters of this study.

### 4.1. Parametric Modeling of Fiber-Reinforced LHPCC

#### 4.1.1. Establishment of the Mesoscale Model

**Figure 18:**
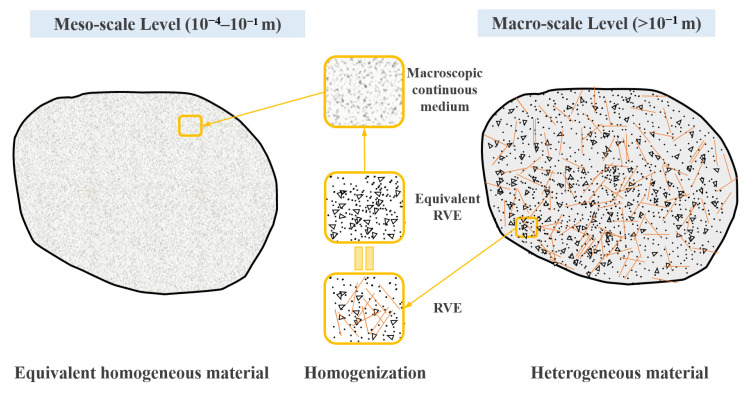
The equivalent transformation between mesoscale and macroscale view in mortar.

There are two types of modeling approaches for fiber-reinforced concrete. One is to model the separated fibers from the cement mortar and assign specific material properties to the fibers, which is common in coarse diameter and large volume steel fiber concrete [52,53]. The other is to consider the fibers and cement mortar as a whole and describe them as fiber-reinforced mortar, common in tiny diameters and large numbers of fibers [54,55]. BF and PVAF used in this paper belong to μm level fibers, and the number of fibers cannot be accurately calculated. Combined with the above analysis, the second modeling approach was adopted in this paper. As shown in Figure 18, in the multi-scale approach, local homogenization of macroscopic solids is usually used to solve the mechanical problems on the mesoscale. The macroscale and the mesoscale are connected by representative volume elements (RVE). The mesoscale mortar consists of pores, fine aggregates, and fibers; a small portion is taken out as RVE; then, the fibers and mortar are equated to the reinforced mortar. The collection of equivalent RVE forms a continuous macroscopic medium that becomes part of the macroscopic equivalent homogeneous material.

**Figure 19:**
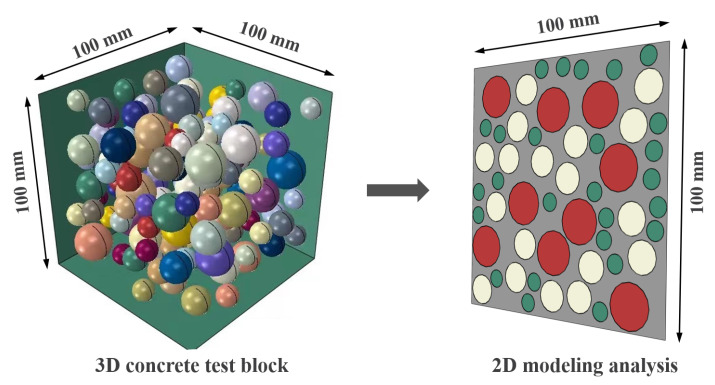
The simplification of concrete specimens from 3D to 2D models.

**Figure 20:**
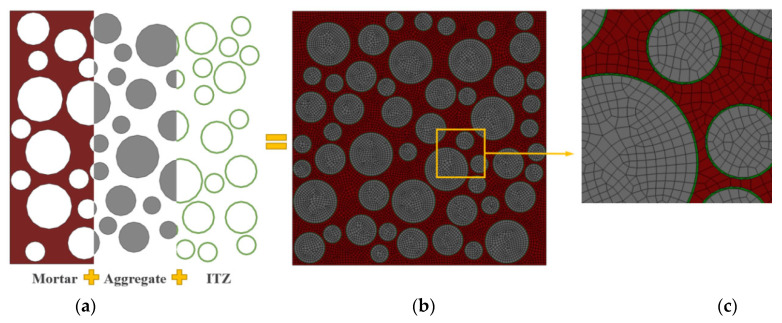
Mesoscale of LHPCC specimen model. (**a**) Three-phase material; (**b**) meshing grid; (**c**) details of the meshing.

In this study, the 3D concrete structure is simplified to a 2D plane for the numerical simulation study, assuming a circular aggregate shape [56,57]. The Monte-Carlo method is used to release the aggregates and generate 2D random aggregate models with dimensions of 100 × 100 mm, as shown in Figure 19. The mesoscale model is established as a three-phase material, as shown in Figure 20. In this case, the aggregate contains three equivalent particle sizes: large stone (particle size *d* = 17 mm), medium stone (*d* = 12 mm), and small stone (*d* = 7 mm), with a total aggregate content of 50%. The green area indicates ITZ, and the interface thickness is set to 0.2 mm, considering the computation amount and combined with the actual ITZ thickness [56,58]. The red area represents the mortar matrix, and the mesh division size is 1 mm [59,60].

#### 4.1.2. Constitutive Relationship of Mesoscale Components of Fiber-Reinforced LHPCC

**Figure 21:**
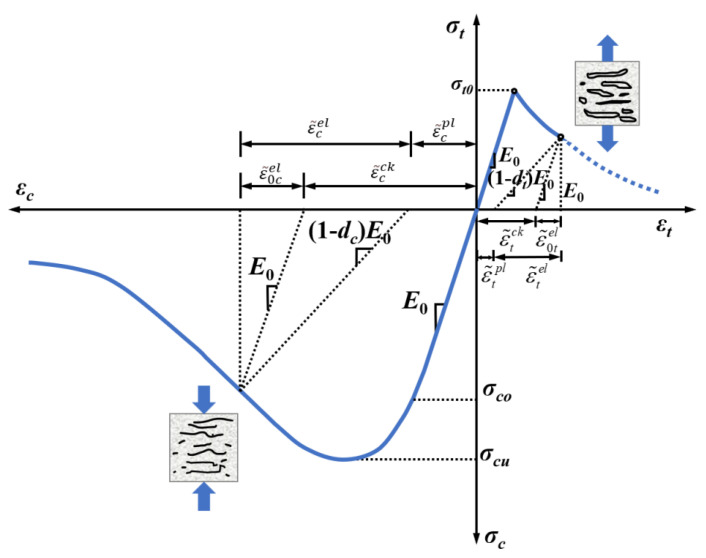
Uniaxial compressive and tensile concrete damage plasticity constitutive model.

Test studies [61,62] have shown that aggregates are generally not damaged under static loading at room temperature, and nonlinear behavior and tensile damage are not considered in this case. Lee et al. [63] proposed a concrete damaged plasticity (CDP) model that can describe the damage characteristics of concrete, which is used in this study to describe the mechanical characteristics of mortar matrix and ITZ due to the similarity of mechanical behavior among concrete, mortar, and ITZ. The CDP models for concrete in uniaxial compression and tension are shown in Figure 21. The core of this failure model assumes that the damage to concrete is in the form of tensile fracture and compression damage, characterized by isotropic damage variables to characterize its stiffness degradation.

(1) $$\sigma =(1-d)D_{0}^{el}:(\varepsilon -\varepsilon^{pl})$$

where, $D_{0}^{el}$ is an isotropic initial undamaged linear elastic tensor; $\varepsilon^{pl}$ is the plastic strain tensor; *d* is a stiffness degradation variable with a value between 0 and 1, where 0 means no damage and 1 means complete damage; *d_t_* and *d_c_* are tensile and compression damage factors, respectively. The stress-strain relationships for the material in uniaxial tensile and compressive states are shown in Equations (2)–(5).

(2) $$\sigma_{t}=(1-d_{t})E_{0}(\varepsilon_{t}-{\tilde{\varepsilon }}_{t}^{pl})$$

(3) $${\tilde{\varepsilon }}_{t}^{pl}={\tilde{\varepsilon }}_{t}^{ck}-\frac{d_{t}}{1-d_{t}}\cdot \frac{\sigma_{t}}{E_{0}}$$

(4) $$\sigma_{c}=(1-d_{c})E_{0}(\varepsilon_{c}-{\tilde{\varepsilon }}_{c}^{pl})$$

(5) $${\tilde{\varepsilon }}_{c}^{pl}={\tilde{\varepsilon }}_{c}^{in}-\frac{d_{c}}{1-d_{c}}\cdot \frac{\sigma_{c}}{E_{0}}$$

where, *σ_c_* and *σ_t_* are the compressive and tensile stresses; *ε_c_* and *ε_t_* are the compressive and tensile strains; *E*_0_ is the initial elastic modulus; ${\tilde{\varepsilon }}_{c}^{pl}$ and ${\tilde{\varepsilon }}_{t}^{pl}$ are the plastic compressive and tensile strain; *d_c_* and *d_t_* are the compressive and tensile damage factors; ${\tilde{\varepsilon }}_{c}^{in}$ and ${\tilde{\varepsilon }}_{t}^{ck}$ are the inelastic strain and cracking strain.

The expansion angle, eccentricity, ratio of biaxial compressive strength to uniaxial compressive ultimate strength, invariant stress ratio, and viscosity parameters in the concrete CDP principal model were taken as 30, 0.1, 1.16, 0.6667, and 0.0005, respectively [64]. In the numerical simulation, due to the relatively large number of divided elements, the damage constitutive model of a relatively simple form is chosen to ensure the efficiency and accuracy of the calculation, provided that the calculation requirements are met. Since the major difference between LHPCC and OPC lies in the composition and the early heat of hydration, there is no significant difference in the damage morphology of the specimens and the tendency of the *σ*-*ε* curve. Therefore, the parameters of the CDP model are determined using the concrete constitutive relations provided by GB50010-2010 [65] in this paper. Among them, the stress-strain relationship for concrete in uniaxial tension is shown in Equations (6)–(9).

(6) $$\sigma_{t}=(1-d_{t})E_{c}\varepsilon_{t}$$

(7) $$d_{t}=\left\{\begin{array}{ccc} 1-\rho_{t}[1.2-0.2x^{5}] & & x\leq 1\\ 1-\frac{\rho_{t}}{\alpha_{t}{\left(x-1\right)}^{1.7}+x} & & x>1 \end{array}\right.$$

(8) $$x=\frac{\varepsilon }{\varepsilon_{t}^{*}}$$

(9) $$\rho_{t}=\frac{f_{t}^{*}}{E_{c}\varepsilon_{c}}$$

where, *α_t_* is the parameter value of the descending stage of the uniaxial stress-strain curve for concrete, take 1.95; $f_{t}^{*}$ is the uniaxial tensile strength of concrete; $\varepsilon_{t}^{*}$ is the peak tensile strain of concrete corresponding to $f_{t}^{*}$, which is taken as 107 × 10^−6^; *d_t_* is the uniaxial tensile damage evolution parameter of concrete.

The stress-strain relationships of concrete in uniaxial compression are shown in (10)–(14).

(10) $$\sigma_{c}=(1-d_{c})E_{c}\varepsilon_{c}$$

(11) $$d_{c}=\left\{\begin{array}{ccc} 1-\frac{\rho_{c}n}{n-1+x^{n}} & & x\leq 1\\ 1-\frac{\rho_{c}}{\alpha_{c}{\left(x-1\right)}^{2}+x} & & x>1 \end{array}\right.$$

(12) $$\rho_{c}=\frac{f_{c}^{*}}{E_{c}\varepsilon_{c}^{*}}$$

(13) $$n=\frac{E_{c}\varepsilon_{c}^{*}}{E_{c}\varepsilon_{c}^{*}-f_{c}^{*}}$$

(14) $$x=\frac{\varepsilon }{\varepsilon_{c}^{*}}$$

where, *α_c_* is the parameter value of the descending stage of the uniaxial compressive stress-strain curve of concrete, which is taken as 1.65; $f_{c}^{*}$ is the uniaxial tensile strength; $\varepsilon_{c}^{*}$ is the corresponding peak compressive strain for $f_{c}^{*}$, taken as 1720 × 10^−6^; *d_c_* is the uniaxial compressive damage evolution parameter of concrete.

**Figure 22:**
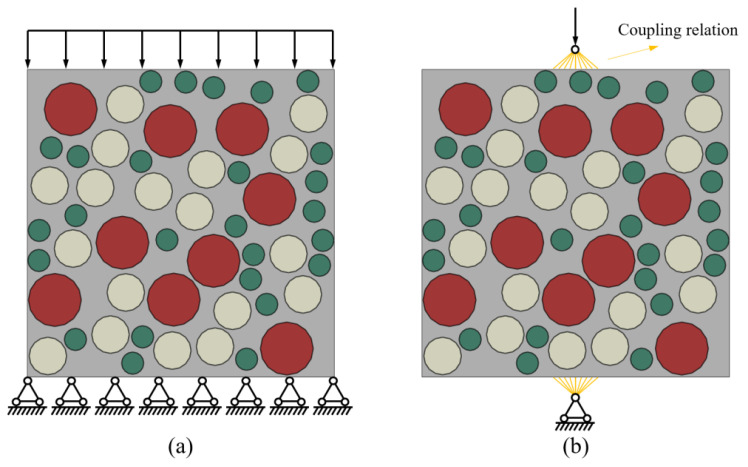
Load application method for compressive and splitting tensile specimens. (**a**) Compressive and (**b**) splitting tensile.

The boundary conditions applied to the compressive and splitting tensile models are shown in Figure 22. The bottom of the compressive model is completely fixed, and the vertical downward load is applied to the upper side of the specimen. The bottom midpoint of the splitting tensile model is fixed, and the vertical downward load is applied at the top midpoint. The upper load and the lower support point are coupled with the top and bottom edges of the specimen. The coupling width is 4 mm for the width of the gasket, and the coupling height is 2 mm for the thickness of the gasket.

#### 4.1.3. Determination of the Mechanical Parameters of the Mesoscale Component

The compressive and tensile simulations were performed on the standard cubic specimens of concrete to determine the mechanical parameters of each mesoscale component, as detailed in Table 12. The ITZ is a mortar matrix material with high porosity, and its mechanical properties can be characterized using weakened mortar matrix mechanical parameters [56]. Since there are few mesoscale simulation studies of LHPCC, the mechanical tests of low-heat Portland cement mortars were used to determine the compressive and tensile strengths as well as fracture energy data. The mechanical properties of the interface phase materials (especially strength parameters) were determined by the trial calculation method. That is, the discounting value of the mortar mechanical parameters is taken, and numerous numerical tests are performed on the compression damage process of the specimen. When the interfacial phase parameters are shown in Table 12, the compressive and tensile mechanical behaviors are in excellent agreement with the test results, suggesting that the values taken for the mechanical parameters of each mesoscale component in the concrete are reasonable.

### 4.2. Mesoscale Simulation of Mechanical Properties of LHPCC

From the study in Chapter 3, it was found that the total content of hybrid fibers in the concrete affected the mechanical properties to a greater extent than the proportion of the two fibers. This chapter focuses on simulating the effect of hybrid fiber content on the compressive and splitting tensile strengths of LHPCC by the mesoscale finite element method, and the simulation results were compared with the test findings.

#### 4.2.1. Uniaxial Compressive Simulation

**Figure 23:**
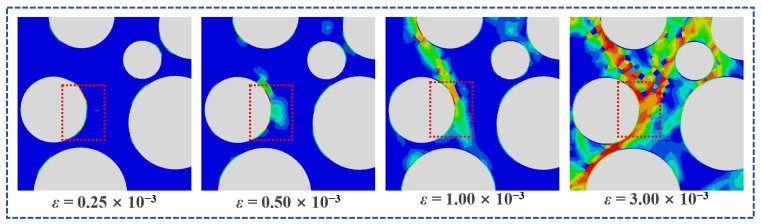
Evolution of ITZ failure under compression.

Figure 23 shows the damage evolution of ITZ under the compression of LHPCC, effectively verifying the actual situation of concrete damage under compression. The cracks first occur in the ITZ and then expand to the surrounding mortar area. The interpenetration of microcracks forms macroscopic damage.

**Figure 24:**
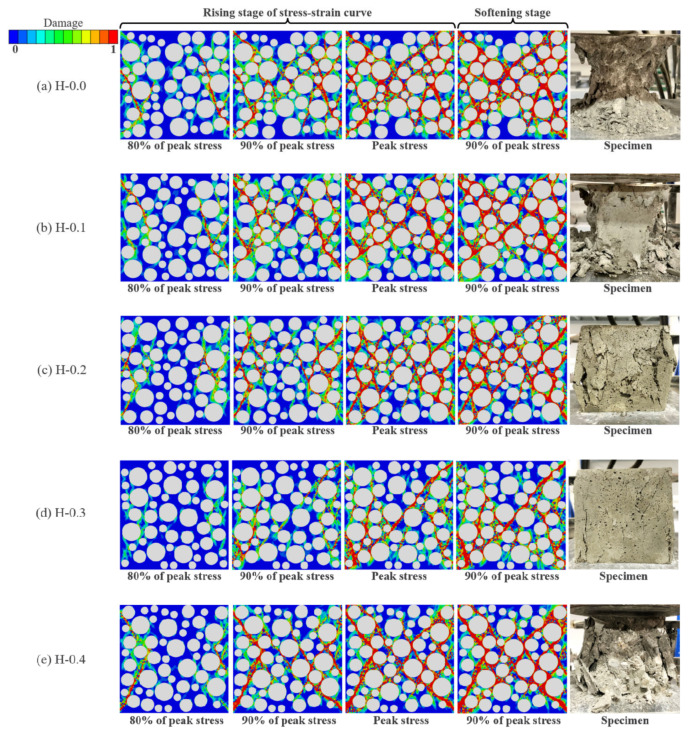
Mesoscale numerical simulation of uniaxial compressive damage evolution of LHPCC with various hybrid fiber contents. (**a**) comparison of the failure model at different loading stages of the concrete without adding fibers; (**b**) comparison of the failure model at different loading stages of the concrete with fiber contents at 0.1%; (**c**) comparison of the failure model at different loading stages of the concrete with fiber contents at 0.2%; (**d**) comparison of the failure model at different loading stages of the concrete with fiber contents at 0.3%; (**e**) comparison of the failure model at different loading stages of the concrete with fiber contents at 0.4%.

Figure 24a–e demonstrates the evolution mechanism of the compressive damage of LHPCC for hybrid fiber contents at 0, 0.1%, 0.2%, 0.3%, and 0.4%. In this study, four stages of the model damage process are taken for analysis: 80%, 90%, and 100% of the peak stress before damage, and 90% of the peak stress after the failure of the concrete. As illustrated in Figure 24a, when there were no fibers in the concrete, the model damage was complete, the damage rate was faster than fiber-reinforced concrete, and the damage path matched highly with the failure pattern of the actual test. When the total fiber content was 0.1% and 0.2%, the specimen damage was decreased to some extent. In Figure 24b,c, the macrocracks decrease, and the damage in the middle of the model gradually weakens relative to the control group when it reaches 80% of the peak stress. The cracks in the center of the specimen gradually appear when it reaches 90% of the peak stress, but the number of cracks decreases relative to the control group. When the peak stress and 90% of the stress after the peak were reached, the model was basically damaged, generating penetration cracks. However, the crack width was reduced in comparison to the control group, and the overall damage was weakened. When the total fiber content reaches 0.3%, as shown in Figure 24d, the number of cracks is significantly reduced in all four stages of damage. The compressed model still maintains a high level of integrity, which is consistent with the damage pattern of the specimens in the mechanical tests. When the total fiber content reaches 0.4%, the high content causes the mechanical properties of mortar to be reduced, and the damage is serious. However, the addition of these fibers still has the characteristics of toughening and crack resistance relative to the control group concrete.

The compressive strength of LHPCC with different hybrid fiber contents was simulated and compared with the average compressive strength obtained from mechanical tests, as shown in Table 13. The simulated data matched well with the test data. The data obtained from the simulations are smaller than the test value, which may be due to the errors caused by the influence of multiple factors such as testing machines, specimen size, friction, and stress concentration in the mechanical tests, which are not considered in the finite element simulations.

**Figure 25:**
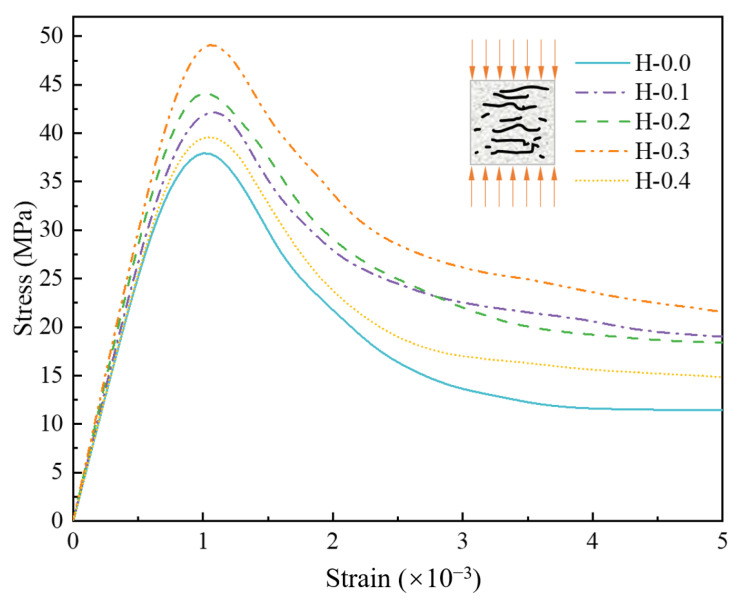
Numerical simulation of compressive stress-strain curve of hybrid fiber-reinforced LHPCC.

The compressive stress-strain curves are depicted in Figure 25. The first 80% of the rising section of all curves are linear, i.e., the damage suffered by the model at this time is recoverable elastic damage. Beyond the critical point of elastic damage, the specimen undergoes plastic deformation until it reaches the peak stress, resulting in the failure of the specimen. During the softening stage, the slopes of the different curves were close to each other, indicating that the change in mortar strength caused by the fibers was not sufficient to affect the rate of decrease in stress. It was found that the elastic modulus was positively correlated with the compressive strength of the specimen when comparing the five curves, i.e., the elastic modulus was the largest in the H-0.3 group and the smallest in the H-0.0 group.

**Figure 26:**
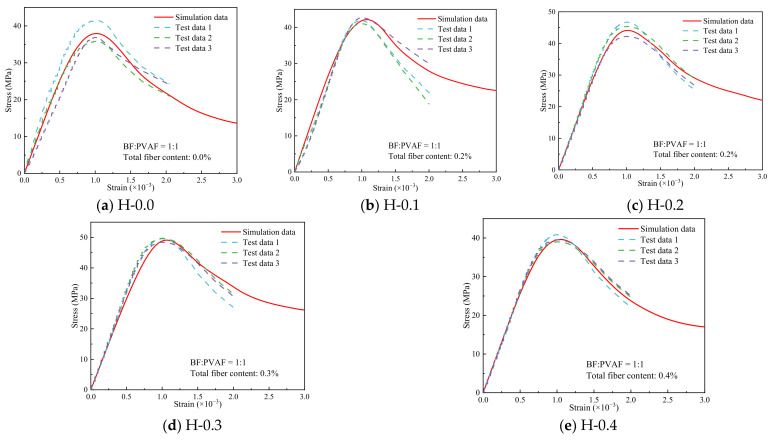
Comparison of compressive stress-strain curves of hybrid fiber-reinforced LHPCC (test and simulation curves).

The stress-strain curves obtained from the tests and simulations were compared, as shown in Figure 26. It can be seen that the curves are in better agreement for different hybrid fiber contents, which indicates that the simulation scheme of this study can verify the test facts. It is worth noting that the slope of the test data is lower than the simulated data in the descending stage of some stress-strain curves, such as in Figure 26b–d. This is because there will be insufficient stiffness of the testing machine in the mechanical test, which causes the derived descending stage to deviate from the actual data. However, the finite element simulation avoids this problem. From Figure 26a,b, it is found that some of the test curves show a concave rise, which is related to internal defects in the concrete and uneven mixing during the blending process. However, the numerical simulation method also does not present such problems.

In summary, the hybrid fiber-reinforced LHPCC compressive mesoscale numerical model established in this section can obtain better simulation results regarding damage characteristics, compressive strength, and stress-strain relationships.

#### 4.2.2. Splitting Tensile Simulation

**Figure 27:**
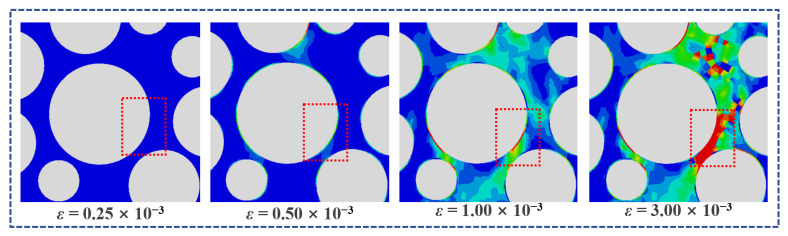
Mesoscale damage evolution of ITZ in the case of splitting tensile.

Similar to the case of uniaxial compressive damage, when the specimen is subjected to splitting tensile damage, cracks are first produced in the ITZ. With the increase in load, the cracks gradually spread around the ITZ, and the cracks penetrate the model, as illustrated in Figure 27.

**Figure 28:**
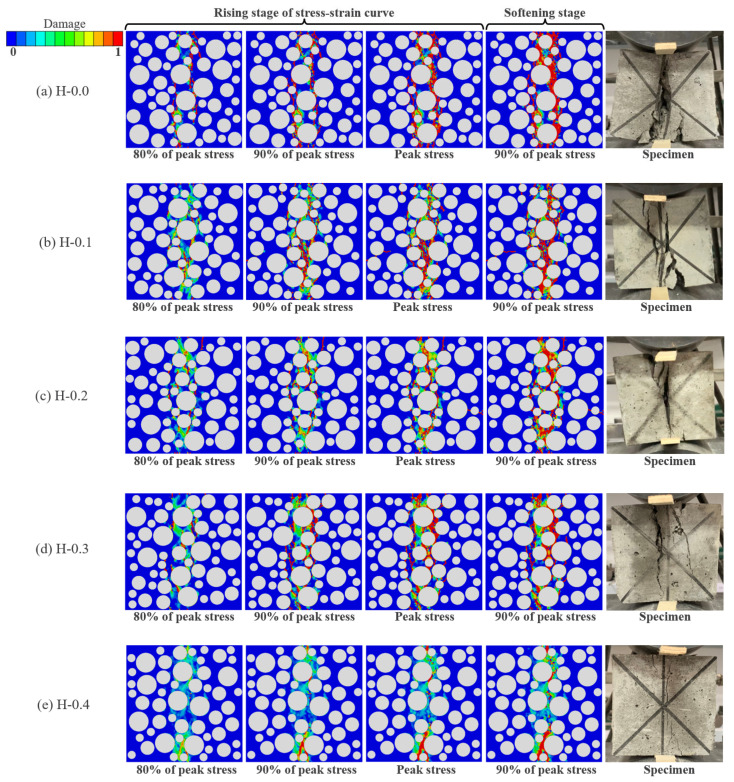
Mesoscale simulation of splitting tensile damage process of LHPCC with different hybrid fiber content. (**a**) comparison of the failure model at different loading stages of the concrete without adding fibers; (**b**) comparison of the failure model at different loading stages of the concrete with fiber contents at 0.1%; (**c**) comparison of the failure model at different loading stages of the concrete with fiber contents at 0.2%; (**d**) comparison of the failure model at different loading stages of the concrete with fiber contents at 0.3%; (**e**) comparison of the failure model at different loading stages of the concrete with fiber contents at 0.4%.

Figure 28 presents the splitting tensile damage of the LHPCC for various fiber contents. In the absence of fiber addition (Figure 28a), the model already produces significant damage when it reaches 80% of the peak stress, and the red macroscopic cracks penetrate the model. After peak stress is reached, the macrocracks will further connect, and the model will be divided into two parts by the cracks, which is consistent with the damage morphology of the tests. As the fiber increases from 0.1% to 0.4%, the damage degree of the model decreases. When the fiber content is 0.1%, it has already produced some toughening and crack-resistance effects on the specimens, but the effect is insignificant. There is little difference in the damage level of the models at 0.2% and 0.3% fiber content, but specimen integrity is significantly improved compared to both the control group and the 0.1% fiber group. When the fiber content reached 0.4%, the cracks were substantially reduced, and some less pronounced macrocracks could be seen after the stress peak. It can be seen that the damage degree of the model decreases with increasing fiber content in the range of fiber content considered in this paper.

**Figure 29:**
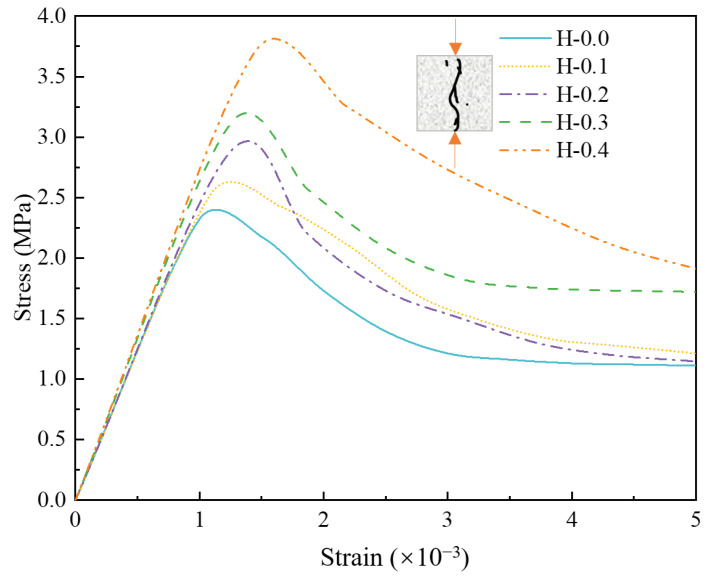
Numerical simulation of splitting tensile stress-strain curve of hybrid fiber-reinforced LHPCC.

Comparing the data on splitting tensile strength obtained from simulation and test, as shown in Table 14, the mesoscale simulation results of this study can correspond well to the mechanical test conclusions. The stress-strain curve obtained from the simulation is shown in Figure 29. The rising section of the curve is basically linear, and the descending section is concave, which is consistent with the constitutive model of this paper.

It can be seen that when the stress was relatively small, the specimens were in the linear elastic stage, and the overall stiffness of the models was significant. In this stage, the stress-strain curves at different fiber contents were basically the same. Significant differences appear in the softening phase after the peak stress. The increase in fiber content causes an increase in ITZ strength and a change in the mesoscale heterogeneity, thus causing a difference in the stress-strain curve at the softening stage. With the further increase in external load, the overall stiffness of the model decreases, the damaged area expands, the splitting crack extends and expands, and the concrete material enters the plastic deformation stage. Finally, the ultimate bearing capacity is exceeded, and brittle damage occurs. Based on the stress-strain diagram, the peak stress and strain increase with the addition of fiber content. This proves that in the range of fiber content in this paper, the higher the content, the stronger the toughening and cracking resistance effect exerted by the fiber. The toughening effect makes the peak stress gradually increase, and the cracking resistance effect makes the peak strain also increase.

**Figure 30:**
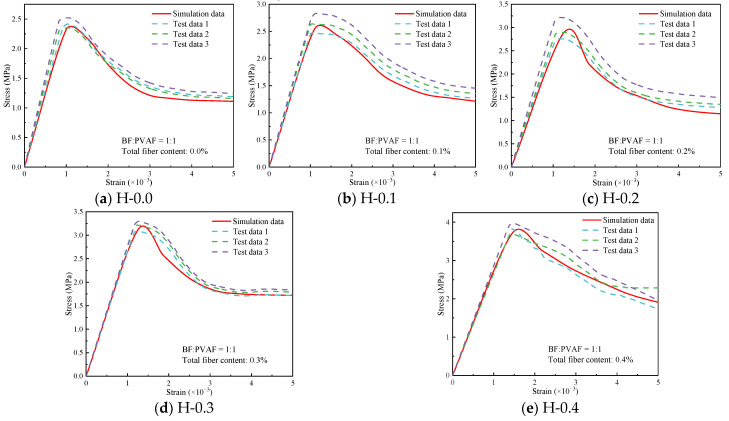
Comparison of splitting tensile stress-strain curves of hybrid fiber-reinforced LHPCC (test and simulation curves).

The stress-strain curves obtained from the finite element simulation were compared with the mechanical test curves, as shown in Figure 30. The rising section of the test and the simulated curve agree well under different hybrid fiber contents. While in the descending section of the curve, since the splitting damage is instantaneous during the test, it is easy to cause the transient change of the loading sensor and the resulting error. The simulated curve is different from the test curve in the descending section but is still within a reasonable range. In summary, this study explains the effects of different hybrid fiber contents on the splitting tensile properties of LHPCC at the mesoscale. Moreover, it verifies the feasibility of the mesoscale numerical simulation method for LHPCC studies.

#### 4.2.3. Effect of Coarse Aggregate Content on the Strength of LHPCC

The aforementioned mesoscale simulation combined the actual test, and the aggregate content was set to 50%. The results were obtained with higher accuracy through the simulation. Therefore, this part keeps all other parameters constant and simulates the effect of aggregate content on the compressive and splitting tensile strengths of hybrid BF and PVAF-reinforced LHPCC to provide a reference for the ratio test study.

**Figure 31:**
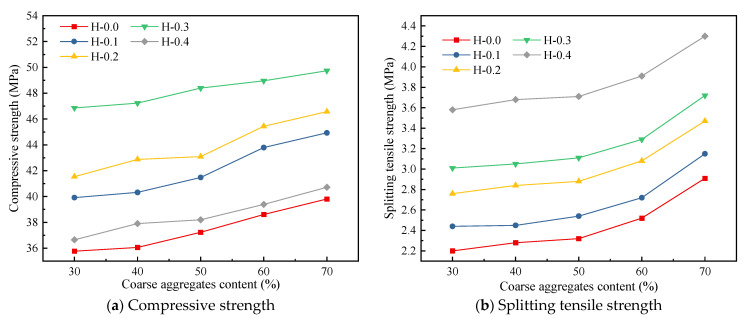
Simulated strength values of hybrid fiber-reinforced LHPCC with different coarse aggregate contents.

In this section, the LHPCC models with aggregate contents of 30%, 40%, 50%, 60%, and 70% are set, and the actual aggregate content of 50% in this paper was used as an intermediate value to investigate the strength differences of LHPCC with different aggregate contents, as shown in Figure 31. With the increase in aggregate content, both the compressive and splitting tensile strengths of LHPCC were enhanced. However, in terms of the degree of influence, the aggregate content had a greater effect on the splitting tensile strength than the compressive strength. For example, as the aggregate content increased from 30% to 70%, the maximum increase in compressive strength was 12.6% for group H-0.1, while the splitting tensile strength had a maximum increase of 32.27% for group H-0.0.

**Figure 32:**
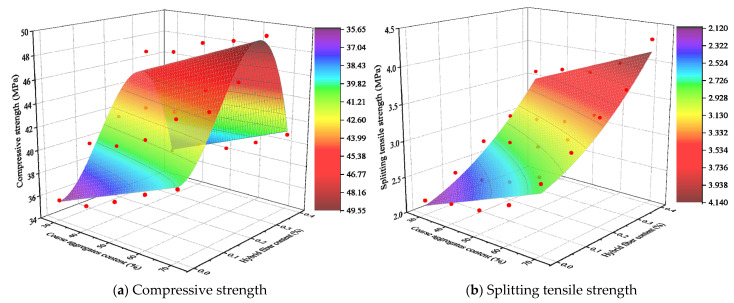
Non-linear surface fitting results for the effect of aggregate and hybrid fiber content on the strength of LHPCC.

The simulation study predicted the common effect of aggregate and hybrid fiber content on the mechanical properties of LHPCC. Therefore, in this paper, a non-linear surface was fitted to the simulation data conclusions to evaluate the effects of the two variables on the compressive and splitting tensile strengths of the material quantitatively through equations. The fitted surface is shown in Figure 32.

Figure 30 corresponds to the equation of compressive strength as shown in Equation (15).

(15) $$\begin{array}{l} f_{c}=0.108x-935.17y^{3}+375.43y^{2}+2.15y+32.42\\ R^{2}=0.91 \end{array}$$

The formula for splitting tensile strength is shown in Equation (16).

(16) $$\begin{array}{l} f_{st}=0.017x+4.69y^{2}+1.48y+1.63\\ R^{2}=0.95 \end{array}$$

where, *f_c_* is the compressive strength of LHPCC and *f_st_* is the splitting tensile strength. *x* denotes the aggregate content (%), and y represents the total hybrid fiber content (%).

The results show that the nonlinear surface fitting models of compressive and splitting tensile strengths of LHPCC can effectively match the simulation results, which is important for the application of hybrid fiber-reinforced LHPCC in hydraulic buildings.

## 5. Conclusions

In this study, mechanical property tests and mesoscale numerical simulations of hybrid BF and PVAF-reinforced LHPCC were conducted to reveal the effect mechanisms of different hybrid fiber contents and the ratio of the two fibers on LHPCC. The major research findings are presented as follows:

- Within the research range of this study, the effect of total fiber content on the slump of fresh concrete is greater than that of different fiber hybrid proportions. When the proportions of the two fibers are 1:1 and the total content is 0.4%, the slump decreases the most, up to 54.55%;
- When BF and PVAF were in equal proportions, the compressive and flexural strengths were greatest when the fiber content was 0.3%, which increased by 29.34% and 35.41% compared to the control group. The splitting tensile strength was greatest when the fiber content was 0.4%, with an increase of 31.59% compared to the control group;
- When the total fiber content was 0.3%, the concrete compressive, splitting tensile, and flexural strengths presented tendencies of ascending and then decreasing as the occupancy of BF increased in the hybrid fiber. The corresponding optimal occupancy of BF was 50%, 75%, and 50%, and the strengths increased by 13.11%, 32.75%, and 16.24% compared with the control concrete;
- The errors in the compressive and splitting tensile strengths of the hybrid BF-PVAF-reinforced LHPCC obtained by numerical simulations and tests are within 5%. The mesoscale model can accurately verify the damage mechanism of concrete specimens, which validates the reliability of the mesoscale numerical model of hybrid BF-PVAF-reinforced LHPCC established in this study.

## 6. Limitations of the Study and Future Research Directions

The study of hybrid BF and PVAF-reinforced LHPCC in this paper has the following basic assumptions: Firstly, the fibers are uniformly distributed in the concrete and in the same direction as the force under external loading. Secondly, the bonding force between the fibers and the concrete is so large that there is no relative slip between them. Although the feasibility of hybrid BF and PVAF to enhance LHPCC was investigated in this paper, it is desirable to properly adjust the basic mix proportion of concrete to obtain more stable properties of LHPCC. As for the study of mesoscale simulation, 2D is less intuitive than the 3D model to reflect the failure mechanism of concrete, which is necessary to be further explored in future studies. In the future, the following aspects can be investigated in depth to further enhance the feasibility of promoting the application of hybrid BF and PVAF-reinforced LHPCC.

- The data on the toughness and ductility of fiber-reinforced concrete are equally necessary, which is crucial to know in hybrid fiber-reinforced LHPCC;
- Concrete materials are brittle and prone to potential safety hazards in hydraulic buildings. Therefore, the fracture properties of hybrid BF and PVAF-reinforced LHPCC should be investigated to overcome the limitations of quasi-brittle fracture of the material;
- The durability study of hybrid BF and PVAF-reinforced LHPCC can be used for life prediction, such as antifreeze, sulfate corrosion resistance, chloride penetration resistance, anti-carbonation, and impermeability properties of concrete materials, for early application in the field of engineering practice.

## Figures and Tables

**Table 1:** Physical properties, mechanical properties, and hydration heat of low-heat Portland cement.

Variety of Cement.	Apparent Density	Standard Consistence	Specific Surface Area	Initial Setting Time	Final Setting Time	Flexural Strength (MPa)		Compressive Strength (MPa)		Hydration Heat (kJ/kg)	
	(g/cm^3^)	(%)	(m^2^/kg)	(min)	(min)	7 d	28 d	7 d	28 d	7 d	28 d
Jiahua P.LH42.5	3.23	25.1	323	236	311	4.7	7.7	21.6	47.3	197	235
Normative standards (GB/T 200-2003)	/	/	≥250	≥60	≤720	≥3.5	≥6.5	≥13.0	≥3.5	≥6.5	≥13.0

**Table 2:** Performance indicators of aggregates.

Types of Aggregates	Aggregate Particle Size (mm)	Fineness Modulus	Apparent Density (kg/m^3^)	Bulk Density (kg/m^3^)	Mud Content (%)	Crush Value (%)	Flat Elongated Particles Content (%)	Porosity (%)
Coarse aggregates	5–20	/	2730	/	0.3	5.9	5.2	41
Fine aggregates	/	2.67	2660	1540	1.6	/	/	36

**Table 3:** Test results of coarse aggregate sieving.

Aggregate Types	Sieve Aperture Size (mm)	Coarse Aggregates	26.5	19	16	9.5	4.75	2.36
		Fine Aggregates	4.75	2.36	1.18	0.6	0.3	0.15
Coarse aggregates	Cumulative sieving residuals (%)		0	23.8	44	75.2	95.8	99.1
	The range of grading required by the specification (%)		0–5	/	30–70	40–80	90–100	90–100
Fine aggregates	Cumulative sieving residuals (%)		0.6	3.1	17.1	49.2	87	93.7
	The range of grading required by the specification (%)		0–10	0–25	10–50	41–70	70–92	90–100

**Table 4:** Quality test results of fly ash.

Varieties of Coal Fly Ash	Fineness (%)	Water Demand ratio (%)	Loss on Ignition (%)	The Content of SO_3_ (%)	Moisture Content (%)	Apparent Density (kg·m^−3^)
Xuanwei Power Plant Level I	6.8	94.05	4.15	0.91	0.14	2430
DL/T 5055-2007	≤12.0	≤95	≤5.0	≤3.0	≤1.0	/

**Table 5:** Performance indicators of BF and PVAF.

Fiber Types	Length (mm)	Diameter (μm)	Density (g/cm^3^)	Tensile Strength (MPa)	Elastic Modulus (GPa)	Fracture Strength (MPa)	Fracture Elongation (%)
BF	12	15	2.65	3200	96	1660	3.2
PVAF	12	40	1.30	1400–1600	35–39	/	17 ± 3.0

**Table 6:** Basic mix proportions (kg/m^3^).

Coarse Aggregates	Fine Aggregates	Cement	Water	Coal Fly Ash	Water-Reducing Agent	Air-Entraining Agent
1292	685	199	130	111	1.53	0.086

**Table 7:** Hybrid fiber content and blending ratio.

*w*/*c*	Fiber Proportion	Total Fiber Content (%)			
	BF: PVAF				
0.47	1:1	0.1	0.2	0.3	0.4
	0:1	0.3			
	1:3	0.3			
	1:1	0.3			
	3:1	0.3			
	1:0	0.3			

**Table 8:** Liquidity test results of fiber-reinforced LHPCC.

Group	Specimen Number	Total Fiber Contents (%)	Contents (%)		Slump (mm)
			BF	PVAF	
Group 1	H-0.0	0.0	0.00	0.00	88
	H-0.1	0.1	0.05	0.05	65
	H-0.2	0.2	0.10	0.10	54
	H-0.3	0.3	0.15	0.15	45
	H-0.4	0.4	0.20	0.20	40
Group 2	H-0-1	0.3	0.000	0.300	69
	H-1-3	0.3	0.075	0.225	59
	H-1-1	0.3	0.150	0.150	51
	H-3-1	0.3	0.225	0.075	47
	H-1-0	0.3	0.300	0.000	43

**Table 9:** Basic mechanical properties of LHPCC with different total hybrid fiber content.

Number	Fiber Content (%)	Compressive Strength (MPa)				Splitting Tensile Strength (MPa)				Flexural Strength (MPa)			
		Test Values	Average Values	Standard Deviation	Coefficient of Variation %	Test Values	Average Values	Standard Deviation	Coefficient of Variation %	Test Values	AVERAGE Values	Standard Deviation	Coefficient of Variation %
H-0.0	0.0	41.50	38.07	2.4689	0.0649	2.41	2.43	0.0704	0.0290	3.88	3.70	0.1314	0.0355
		36.90				2.35				3.65			
		35.80				2.52				3.57			
H-0.1	0.1	41.80	42.51	1.6111	0.0379	2.46	2.64	0.1470	0.0558	3.98	4.15	0.1219	0.0294
		40.99				2.63				4.21			
		44.74				2.82				4.26			
H-0.2	0.2	45.32	44.74	1.8983	0.0424	2.75	2.95	0.2351	0.0797	4.15	4.29	0.1003	0.0234
		46.72				2.89				4.38			
		42.18				3.21				4.34			
H-0.3	0.3	48.41	49.24	0.5938	0.0121	3.08	3.19	0.0865	0.0271	4.95	5.01	0.0920	0.0184
		49.57				3.21				5.14			
		49.75				3.29				4.94			
H-0.4	0.4	39.52	39.78	0.7779	0.0196	3.82	3.83	0.1186	0.0310	4.19	4.12	0.0497	0.0121
		38.99				3.69				4.09			
		40.84				3.98				4.08			

Note, the number “H” stands for Hybrid.

**Table 10:** Basic mechanical properties of LHPCC with different fiber hybrid ratios (total fiber content: 0.3%).

Number	Fiber Proportion	Compressive Strength (MPa)				Splitting Tensile Strength (MPa)				Flexural Strength (MPa)			
	BF: PVAF	Test Values	Average Values	Standard Deviation	Coefficient of Variation %	Test Values	AVERAGE Values	Standard Deviation	Coefficient of Variation %	Test Values	Average Values	Standard Deviation	Coefficient of Variation %
H-0-1	0:1	42.86	43.41	0.6034	0.0139	2.68	2.87	0.1359	0.0473	4.25	4.31	0.0432	0.0100
		44.25				2.94				4.33			
		43.12				2.99				4.35			
H-1-3	1:3	51.68	47.74	2.8611	0.0599	3.11	3.02	0.0638	0.0211	4.39	4.50	0.0787	0.0175
		46.55				2.97				4.57			
		44.98				2.98				4.54			
H-1-1	1:1	49.96	49.10	1.3821	0.0281	3.08	3.19	0.0865	0.0271	4.95	5.01	0.0920	0.0184
		47.15				3.21				5.14			
		50.19				3.29				4.94			
H-3-1	3:1	45.95	43.49	2.1512	0.0495	3.71	3.81	0.0779	0.0204	4.74	4.83	0.0779	0.0161
		43.81				3.9				4.93			
		40.71				3.82				4.82			
H-1-0	1:0	41.22	40.79	0.7316	0.0179	2.45	2.64	0.1417	0.0537	4.78	4.62	0.1203	0.0260
		39.76				2.68				4.59			
		41.39				2.79				4.49			

Note, the number “H” stands for Hybrid.

**Table 11:** Codes predicted mechanical properties of normal concrete.

Mechanical Properties	ACI 318-11 [46]	Eurocode 2 [47]
Splitting tensile strength, *f_st_* (MPa)	$f_{st}=0.53\cdot \sqrt{f_{c}^{\prime }}$	$f_{st}=0.3\cdot f_{c}^{\prime 2/3}$
Flexural strength, *f_r_* (MPa)	$f_{r}=0.62\cdot \sqrt{f_{c}^{\prime }}$	$f_{r}=0.435\cdot f_{c}^{\prime 2/3}$

where, *f_c_′* is the characteristic compressive strength.

**Table 12:** Mesoscale each components mechanical parameters of hybrid fiber-reinforced LHPCC.

Parameters	Aggregates Phase	Mortar Phase: Fiber Content					Interface Phase
		0	0.1%	0.2%	0.3%	0.4%	
Young’s modulus, *E* (GPa)	50 ^1^	39.1 ^2^	44 ^2^	46.3 ^2^	50.2 ^2^	40.3 ^2^	26 ^3^
Poisson’s ratio, *ν*	0.16 ^1^	0.2 ^1^	0.2 ^1^	0.2 ^1^	0.2 ^1^	0.2 ^1^	0.22 ^3^
Compressive strength, *σ_c_* (MPa)	130 ^1^	42.8 ^2^	48.3 ^2^	49.8 ^2^	55.3 ^2^	45.4 ^2^	27.5 ^4^
Tensile strength, *σ_t_* (MPa)	10 ^1^	2.4 ^2^	2.6 ^2^	3.0 ^2^	3.2 ^2^	3.8 ^2^	2.75 ^4^

where, “^1^” refer to the paper Jin et al. [66], “^2^” are obtained from mechanical tests, “^3^” refer to the paper by Du et al. [67], and “^4^” are obtained from trial calculations.

**Table 13:** Comparison of test and simulated compressive strength values of hybrid BF-PVAF-reinforced LHPCC.

Group	Compressive Strength (MPa)		Difference Between Test and Simulation (MPa)	Error Relative to the Test Value (%)
	Test Data	Simulation Data		
H-0.0	38.07	37.23	0.84	2.21
H-0.1	42.51	41.48	1.03	2.42
H-0.2	44.74	43.09	1.65	3.69
H-0.3	49.24	48.40	0.84	1.71
H-0.4	39.78	38.20	1.58	3.97

**Table 14:** Comparison of test and simulated splitting tensile strength values of hybrid BF-PVAF-reinforced LHPCC.

Group	Splitting Tensile Strength (MPa)		Difference Between Test and Simulation (MPa)	Error Relative to the Test Value (%)
	Test Data	Simulation Data		
H-0.0	2.43	2.32	0.11	4.53
H-0.1	2.64	2.54	0.10	3.79
H-0.2	2.95	2.88	0.07	2.37
H-0.3	3.19	3.11	0.08	2.51
H-0.4	3.83	3.71	0.12	3.13

## Data Availability

The data that has been used are confidential.

## Abbreviations

BF	Basalt fiber
PVAF	Polyvinyl alcohol fiber
LHPCC	Low-heat Portland cement concrete
OPC	Ordinary Portland cement
HFRC	Hybrid fiber-reinforced concrete
ITZ	Interface transition zone
RVE	Representative volume elements
CDP	Concrete damaged plasticity
